# Bifacial cambium stem cells generate xylem and phloem during radial plant growth

**DOI:** 10.1242/dev.171355

**Published:** 2019-01-09

**Authors:** Dongbo Shi, Ivan Lebovka, Vadir López-Salmerón, Pablo Sanchez, Thomas Greb

**Affiliations:** 1 Department of Developmental Physiology, Centre for Organismal Studies (COS), Heidelberg University, Im Neuenheimer Feld 230, 69120 Heidelberg, Germany; 2 Gregor Mendel Institute (GMI), Austrian Academy of Sciences, Vienna Biocenter (VBC), Dr. Bohr-Gasse 3, 1030 Vienna, Austria

**Keywords:** Stem cells, Secondary growth, Cambium, Cell lineage, Meristem, *Arabidopsis thaliana*

## Abstract

A reduced rate of stem cell division is considered a widespread feature which ensures the integrity of genetic information during somatic development of plants and animals. Radial growth of plant shoots and roots is a stem cell-driven process that is fundamental for the mechanical and physiological support of enlarging plant bodies. In most dicotyledonous species, the underlying stem cell niche, the cambium, generates xylem inwards and phloem outwards. Despite the importance and intriguing dynamics of the cambium, the functional characterization of its stem cells is hampered by the lack of experimental tools for accessing distinct cambium sub-domains. Here, we use the hypocotyl of *Arabidopsis thaliana* to map stem cell activity in the proliferating cambium. Through pulse labeling and genetically encoded lineage tracing, we find that a single bifacial stem cell generates both xylem and phloem cell lineages. This cell is characterized by a specific combination of *PXY* (*TDR*), *SMXL5* and *WOX4* gene activity and a high division rate in comparison with tissue-specific progenitors. Our analysis provides a cellular fate map of radial plant growth, and suggests that stem cell quiescence is not a general prerequisite for life-long tissue production.

This article has an associated ‘The people behind the papers’ interview.

## Abstract (precis)

**Highlighted Article:** A single bifacial stem cell that is characterized by the combined activity of *PXY* (*TDR*), *SMXL5* and *WOX4* genes generates both wood and bast during radial plant growth.

## INTRODUCTION

**Fig. 1.**
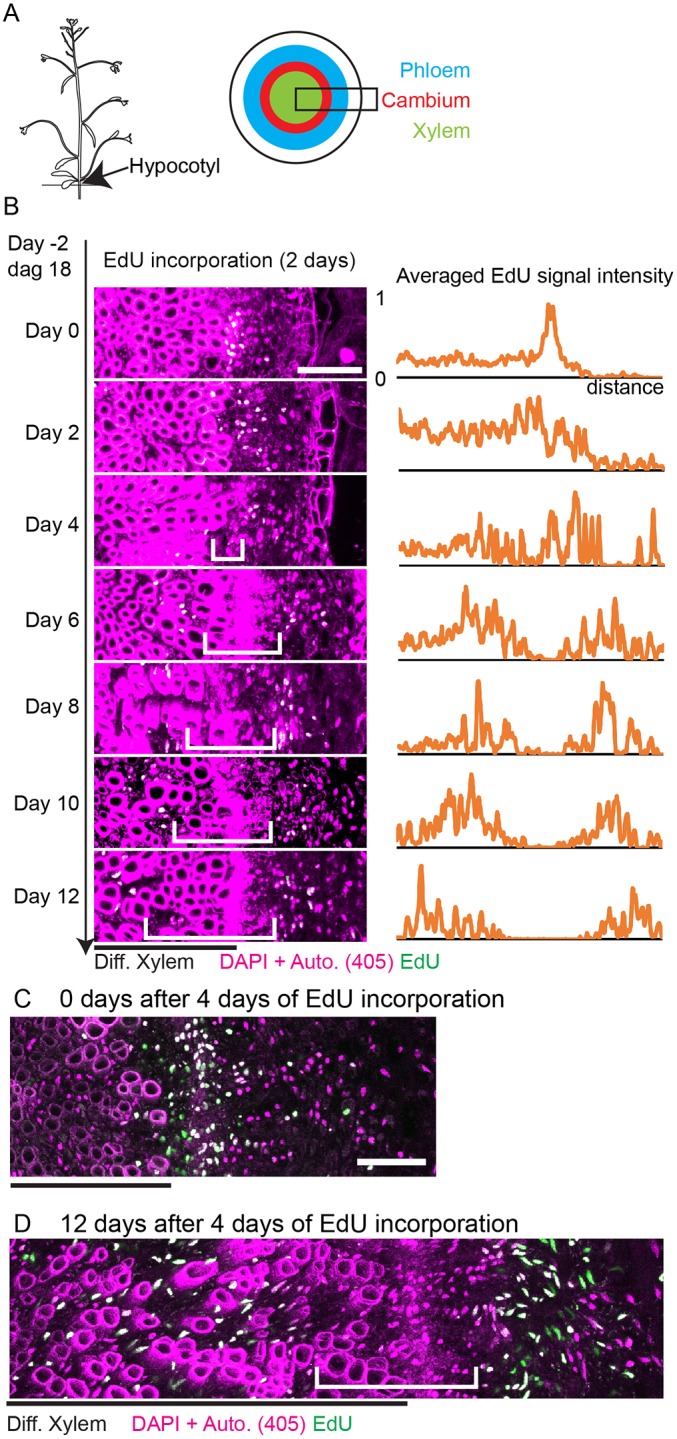
**Pulse-chase EdU assay during radial growth of hypocotyl.** (A) Schematic of a hypocotyl cross section. The black frame indicates the region analyzed in this study. (B) Maximum intensity projection of confocal images of cross sections of EdU-incubated hypocotyls (left) and the averaged EdU signal intensity profile (right). The averaged profile was obtained from multiple images and the numbers of images are *n*=8 (Day 0), *n*=6 (Day 2), *n*=4 (Day 4), *n*=6 (Day 6), *n*=6 (Day 8), *n*=6 (Day 10), *n*=4 (Day 12). The cross-correlation values were lower than 0.3 in many pair-wise comparisons, suggesting that the profiles were different from each other. For details, see the Intensity profile analysis section in Materials and Methods. See Table S1 for cross correlation analysis. (C,D) Hypocotyl sections images of 4 days EdU incorporation. *n*=5 each. Black line indicates the region of differentiated xylem; white brackets indicate the continuous region without strong EdU signal; EdU staining is shown in green. Nuclei were stained with DAPI and the cell wall of differentiated xylem are visualized in the same magenta channel because of the autofluorescence excited by the 405 nm laser. As most of the EdU-positive nuclei are also stained by DAPI, the color appears to be white in the merged picture. Some green nuclei observed in C and D are visible because of the weaker penetration of DAPI staining compared with EdU staining. Scale bars: 50 µm.

After the initial development which forms the primary growth axes, many plants expand their girth by a process called radial, or secondary, growth. Radial growth is fundamental for plant growth, as it provides long-distance transport capacities for nutrients and water (xylem) and assimilates (phloem) for the enlarging plant body, and it is the most important process for terrestrial biomass accumulation (Bar-On et al., 2018). In the predominant dicotyledonous species, expansion is achieved through the bidirectional production of the vascular tissues, xylem (wood) and phloem (bast), by the proliferating cambium (Fig. 1A). Besides its functional importance, the cambium is considered an instructive model for stem cell biology in plants and beyond (Greb and Lohmann, 2016; Johnsson and Fischer, 2016; Shi et al., 2017).

The cellular view of cambium stem cell activity is mostly based on a long history of classical histological analyses (Larson, 1994; Sankar et al., 2014). In these approaches, proliferative cells are identified based on their smaller shape compared with neighboring cells and their lack of signs of specialization. The most popular view on cambium dynamics was brought forward almost 150 years ago by the botanist Karl Gustav Sanio, who postulated the existence of one single stem cell in each radial cell file, which alternates between the production of xylem and phloem precursors (Sanio, 1873), a view which has been shared by most authors since (Rothert and Jost, 1934). Independent from favoring this so-called uniseriate model over a multiseriate model, which postulates the existence of multiple stem cells in radial orientation, the cambium is defined as the region of dividing cells which includes cambial initials (stem cells) and phloem or xylem mother cells (progenitor cells) (Butterfield, 1975).

However, stem cell activity has not been mapped experimentally because of the lack of unambiguous molecular markers for cambium stem cells and the lack of possibility for live-cell imaging. It is still unclear, therefore, whether a single stem cell can serve as the source for both phloem and xylem (Mazur and Kurczynska, 2012; Murmanis, 1970) and whether there is a quiescent center in the cambium region, as it is found in shoot and root apical meristems that generate the whole above- and below-ground body parts, respectively (Greb and Lohmann, 2016). This lack of experimental access is an obstacle to a deeper understanding of the molecular regulation of radial plant growth and the evolution of plant stem cell systems (Johnsson and Fischer, 2016).

Here, we characterize cambium dynamics by focusing on the radial expansion of the hypocotyl of *Arabidopsis thaliana* as a well-established experimental model for radial plant growth (Lehmann and Hardtke, 2016). In particular, we establish different transgenic markers that define a proximal, a central and a distal cambium domain. We further reveal that the proximal domain represents a site of xylem formation and the distal cambium domain contains cells that are determined for phloem development. Intriguingly, we identify a narrow domain in the cambium center, which contains bifacial strongly proliferating stem cells that feed both xylem and phloem production.

## RESULTS AND DISCUSSION

### Proliferative cells predominantly localize to a single domain in the cambium area

To map proliferative cambium cells and their fate, we first carried out a pulse-labeling experiment using the thymidine analogue 5-ethynyl-2′-deoxyuridine (EdU) (Chehrehasa et al., 2009). For this, *Arabidopsis* seedlings were transferred to liquid medium that was supplemented with EdU 18 days after germination (dag). Two days later, plants were transferred to soil and cross sections from hypocotyls were analyzed at different times after the transfer. These analyses showed that, immediately after EdU incubation, EdU-positive nuclei were mostly present in one domain immediately distal to the differentiated xylem (Fig. 1B), which suggested that only those cells had replicated their DNA during the incubation period. Two days after EdU incubation, EdU-positive nuclei were detected in one slightly larger region that included differentiated xylem cells (Fig. 1B). From day 4 to day 12, EdU-positive nuclei were clearly separated in two domains, one in the differentiated xylem and one, more distal, containing differentiated phloem cells (Fig. 1B). Over time, the distance between the two domains increased, which demonstrated that new cells were produced continuously in the cambial area and that previously produced descendants were left behind in the case of xylem cells, which keep their position within the hypocotyl, or pushed toward the organ periphery in the case of phloem cells. These results support the classical view on radial plant growth, in which cells in the cambium region proliferate and provide cells for vascular tissue production bidirectionally. Importantly, there was no indication of slowly dividing cells in the cambium center retaining EdU labeling, as was found in the centers of apical meristems (Watson et al., 2016). To challenge this conclusion, we increased the duration of EdU incorporation to 4 days thereby raising the penetrance of nucleus labeling (Fig. 1C), and 12 days after the incorporation, we again could not find EdU-positive nuclei in the cambium region (Fig. 1D). This suggested that quiescence of cambium stem cells is not a prominent feature within the process of radial plant growth.

### *PXY*, *SMXL5* and *WOX4* gene activities define three cambium domains

**Fig. 2.**
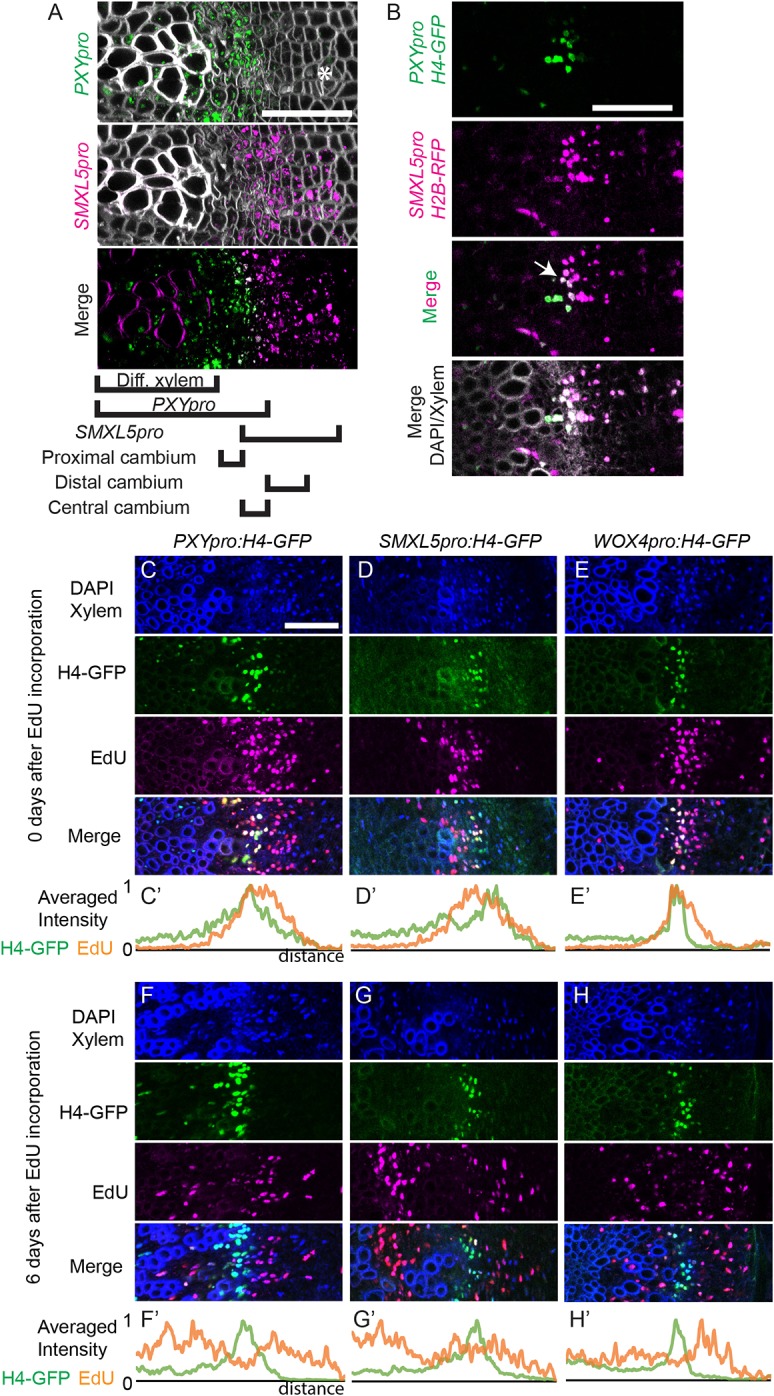
**Characterization of *PXY*, *SMXL5* and *WOX4* promoter activities.** (A) Maximum intensity projection of confocal images of *PXY_pro_:CFP;SMXL5_pro_:YFP* hypocotyl cross sections at 22 dag. Direct Red 23 cell wall staining is shown as white [note that the magenta signal in the xylem cell wall of the merged image is because of Direct Red 23 staining, which can be weakly excited by the 514 nm laser (YFP channel). We could not detect such a signal without staining (Fig. S1)]. Asterisk indicates phloem cells. (B) Confocal images of *PXYpro:H4-GFP;SMXL5pro:H2B-RFP* hypocotyl cross sections at 22 dag. Nuclei (DAPI) and xylem cell wall (auto-fluorescence) are shown in white in the bottom image. Arrow indicates nuclei with both *PXY_pro_* and *SMXL5_pro_* activity. *n*=2. (C-H) Maximum intensity projection of confocal images of *PXY_pro_:H4-GFP, SMXL5_pro_:H4-GFP, WOX4_pro_:H4-GFP* hypocotyl cross sections after EdU incorporation. (C′-H′) Averaged EdU and H4-GFP signal intensity profile are obtained from multiple images and normalized to 1. *n*=12 (C), *n*=8 (D), *n*=10 (E), *n*=9 (F), *n*=7 (G) and *n*=12 (H). The hypocotyl center is located to the left. Scale bar: 50 µm.

For associating cell proliferation with distinct cambium domains, we first characterized promoter activities of the cambium-related *PHLOEM INTERCALATED WITH XYLEM* (*PXY*) and *SUPPRESSOR OF MAX2 1-LIKE PROTEIN 5* (*SMXL5*) genes (Agusti et al., 2011b) using a transgene carrying reporters that expressed different fluorescent proteins that were targeted to the endoplasmic reticulum (ER) under the control of each promoter [*PXY_pro_:CYAN FLUORESCENT PROTEIN;SMXL5_pro_:YELLOW FLUORESCENT PROTEIN* (*PXY_pro_:CFP;SMXL5_pro_:YFP*)]. As indicated before (Brackmann et al., 2018; Fisher and Turner, 2007; Gursanscky et al., 2016; Hirakawa et al., 2008; Kondo et al., 2014), the activity of the *PXY* promoter was detected in the cambium region and partly in differentiated xylem cells (Fig. 2A, Fig. S1). In contrast, the activity of the phloem-related *SMXL5* promoter (Wallner et al., 2017) was detected in a domain that was distal to *PXY*-positive cells, which contained undifferentiated cambium cells and differentiated phloem (Fig. 2A, Fig. S1). Interestingly, both promoter activities slightly overlapped at the margin of both activities in one to two cells in radial orientation (Fig. 2A,B). Based on these observations, we designated the *PXY*-positive cambium domain as ‘proximal’, the *SMXL5*-positive domain as ‘distal’, and the domain displaying both activities as ‘central’.

To see how cell proliferation is distributed relative to these domains, we generated reporter plant lines in which a protein fusion of histone H4 and the green fluorescent protein (H4-GFP) was expressed under the control of the *PXY* or the *SMXL5* promoter. When carrying out EdU-based pulse labeling with these lines we observed that, immediately after 4 days of EdU incubation, *PXY_pro_*-positive nuclei, and nuclei from cells located distal to those, showed EdU incorporation (Fig. 2C). In comparison, *SMXL5_pro_*-positive nuclei, as well as nuclei proximal to those, were EdU-positive (Fig. 2D). We also performed the same analysis using the *WUSCHEL RELATED HOMEOBOX4* (*WOX4*) promoter, which is active in the cambium and in young differentiated xylem cells (Hirakawa et al., 2008; Suer et al., 2011), and whose activity fully overlaps with the *PXY* promoter activity (Brackmann et al., 2018) (Fig. S2). As expected, results of EdU incorporation assays using the *WOX4* reporter line recapitulated the results obtained with the *PXY* promoter line (Fig. 2E), which demonstrated the robustness of our analysis. Six days after EdU incubation, most of the *SMXL5_pro_*-, *PXY_pro_-* and *WOX4_pro_*-positive nuclei which had initially been EdU-positive were EdU-negative, but were encompassed by two peaks of EdU-labeled nuclei (Fig. 2F-H). This observation suggested that DNA replication had occurred around the central cambium during these 6 days. Taken together, these observations indicate that cell proliferation is found in the proximal, the central and the distal cambium domain, defined by different promoter activities.

### Cell lineage tracing in distinct cambium domains identifies bifacial stem cells

**Fig. 3.**
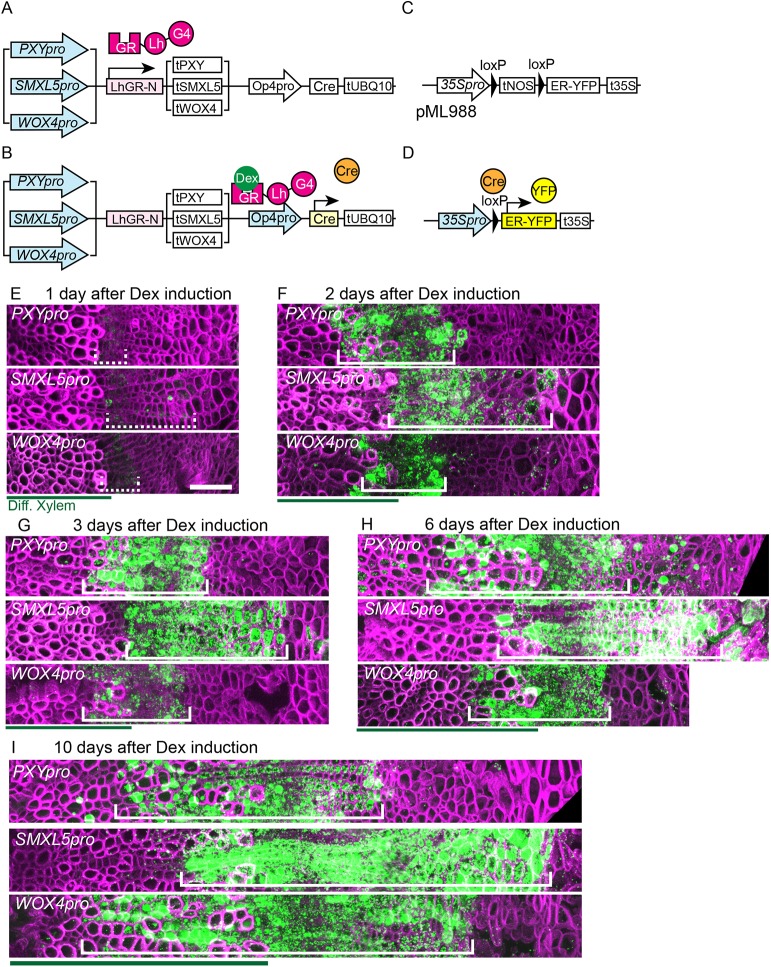
**Cell lineage tracing of *PXY*-, *WOX4*- and *SMXL5*-positive cells.** (A-D) The scheme of the genetically encoded cell lineage tracking system. (E-I) Maximum intensity projection of confocal images of cross sections of Dex-induced hypocotyls, *PXY_pro_:LhGR-N;Op4_pro_:Cre;;pML988* (*PXY_pro_*), *SMXL5_pro_:LhGR-N;Op4_pro_:Cre;;pML988* (*SMXL5_pro_*) and *WOX4_pro_:LhGR-N;Op4_pro_:Cre;pML988* (*WOX4_pro_*). YFP activity is shown in green and cell walls are visualized by Direct Red 23 staining (magenta). Images of 1 day (E), 2 days (F), 3 days (G), 6 days (H) and 10 days (I) after Dex induction are shown. Green lines indicate the region of differentiated xylem; white brackets indicate the region with continuous YFP activity in radial orientation; dashed white brackets indicate respective expression domains. The hypocotyl center is located on the left. *n*=3, 4, 2 (E), *n*=2, 3, 3 (F), *n*=2, 5, 3 (G), *n*=2, 5, 3 (H) and *n*=2, 5, 2 (I). Uncropped images are shown in Fig. S3. Scale bar: 50 µm.

With the aim of tracking the fate of cells that were located in the proximal, central and distal cambium domains, we generated a genetically encoded system for cell lineage tracing based on the Cre-LoxP system (Sternberg and Hamilton, 1981). In particular, we generated a three-component system in which a dexamethasone (Dex)-inducible protein fusion of the ligand binding domain of the glucocorticoid receptor (GR) and the LhG4 transcription factor (LhGR-N) (Craft et al., 2005) was expressed under the control of the *PXY*, *SMXL5* or the *WOX4* promoter (Fig. 3A). In our case, the LhGR-N initiated the expression of the Cre recombinase that induced genomic recombination at LoxP sites, bringing the ER-YFP reporter under the control of the constitutive CaMV 35S promoter (Odell et al., 1985) (Fig. 3B,C). ER-YFP was therefore expressed constantly in *PXY_pro_*-, *WOX4_pro_*- and *SMXL5_pro_*-positive cells and in all their derivatives, respectively (Fig. 3D).

To determine clonal relationships of *PXY_pro_*-, *WOX4_pro_*- and *SMXL5_pro_*-positive cells, we first applied a high dose of Dex locally to the hypocotyl and analyzed hypocotyls at different times after the induction (Fig. 3E-I, Fig. S3). One day after induction, YFP levels were weak, but recapitulated the expression pattern of the *PXY*, the *WOX4* and the *SMXL5* promoter, respectively (Fig. 3E, Fig. S3). Two days after induction, high levels of YFP activity were found in continuous regions along the radial axis (Fig. 3F, Fig. S3). These regions extended radially in two directions over a period of 10 days after the induction (Fig. 3G-I, Fig. S3) suggesting that vascular cells generated by the cambium during radial growth originate from cells that are characterized by all three promoter activities. In contrast, cells positive for the *NAC SECONDARY WALL THICKENING PROMOTING FACTOR 3* (*NST3*) promoter that is active in the differentiated xylem (fibers) (Mitsuda et al., 2007), or for the *ALTERED PHLOEM DEVELOPMENT* (*APL*) promoter that marks differentiated phloem (Bonke et al., 2003), gave rise exclusively to xylem and phloem, respectively (Fig. S4A-H). When comparing the dynamics of size changes of the YFP-positive domains we found that, in *PXY* and *WOX4* promoter lines, these domains extended into the differentiated xylem 2 days after induction (Fig. 3F). In contrast, in *SMXL5* promoter lines the YFP-positive domain included xylem cells only from 6 days onwards (Fig. 3H). These differences suggest that cells in the proximal cambium differentiate into xylem earlier than cells located in the central or distal domain. Because only the central domain is characterized by the combined activity of the *PXY* and the *SMXL5* promoter (Fig. S4I), we concluded that this domain harbors common stem cells for xylem and phloem formation.

### Cell lineage analysis of *PXY_pr_*_o_, *SMXL5_pro_* and *WOX4_pro_* positive cells

**Fig. 4.**
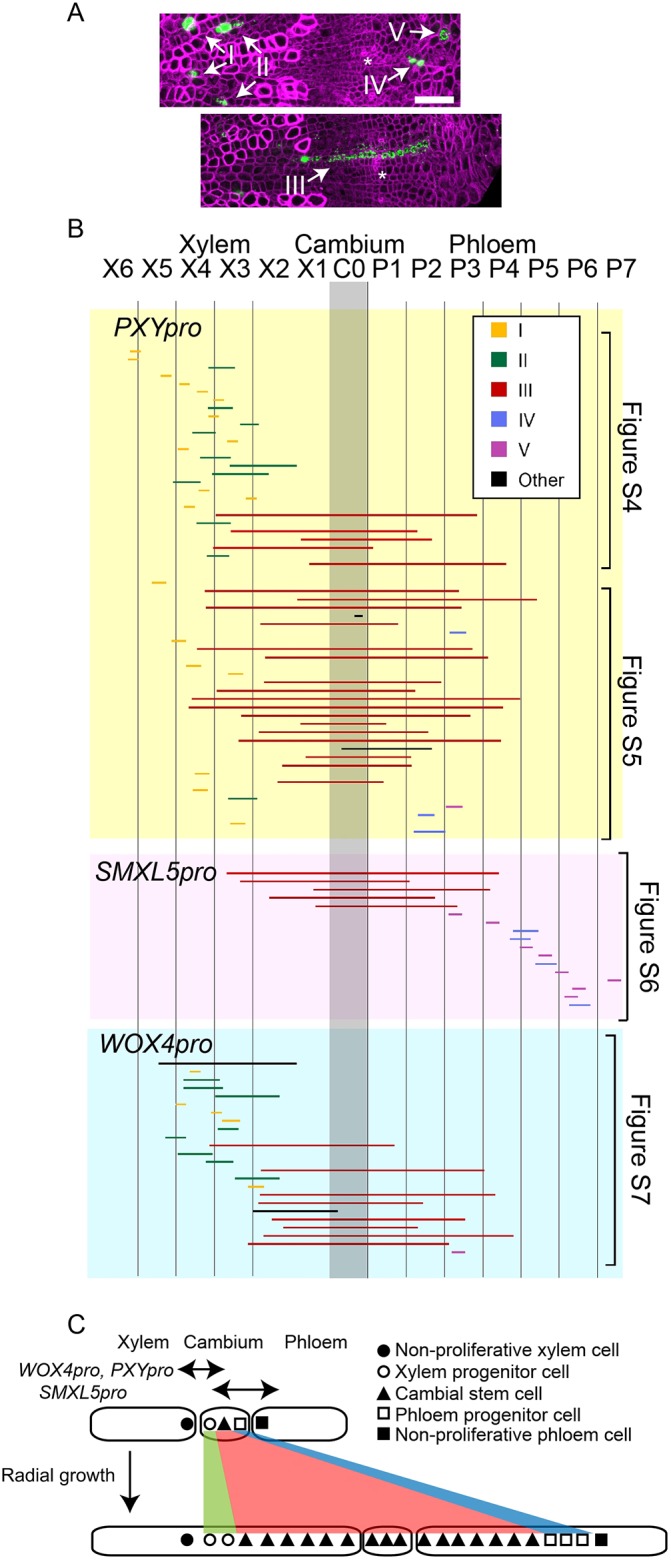
**Analysis of individual cell clones.** (A) Maximum intensity projection of confocal images from cross sections of hypocotyls showing the representative morphology clone types at 10 days after Dex induction. YFP activity is shown in green and cell walls are stained by Fast Red 23 (magenta). Asterisks indicate phloem poles. (B) Summary of cell clone morphology obtained 10 days after Dex induction to *PXY_pro_:LhGR-N;Op4_pro_:Cre;;pML988* (*PXY_pro_*), *SMXL5_pro_:LhGR-N;Op4_pro_:Cre;;pML988* (*SMXL5_pro_*) and *WOX4_pro_:LhGR-N;Op4_pro_:Cre;;pML988* (*WOX4_pro_*). The boundary between differentiated xylem and the cambium was used to align images. A grid is shown with 50 µm intervals. Clone extension is visualized in lines with colors according to their morphology types. Micrographs that correspond to each clone are shown in Figs S5-S8. (C) A schematic cell fate map during 10 days of radial hypocotyl growth. Cells that are indicated in the bottom scheme originate from cells that are indicated with the same character in the top scheme. Scale bar: 50 µm.

Because it was not possible to track the fate of individual cells using a thorough induction of the Cre recombinase in the respective domains, we generated single-cell clones by reducing the time and the dosage of Dex application. When analyzing hypocotyls 10 days after Dex induction, in total we identified 99 YFP-positive clones in the cambium or in cambium-derived tissues (Fig. 4A,B, Figs S5-S8). As a large majority (73/99) of those clones consisted of individual files in radial orientation, at least along the cambium area, we estimated that single cells were the origin of at least 74% of those clones. This conclusion is based on the notion that clones including more than one cell file would have been observed more often if neighboring cells were frequently induced. Our calculation can also be considered an underestimation, because the few clones that span more than one radial cell file (26/99) may have originated from anticlinal divisions of one YFP-positive cell after Cre-induced recombination. Based on their morphology and localization, 95 of the 99 clones (96%) could be categorized into five types: (I) single-cell clones in the xylem region; (II) multiple-cell clones in the xylem; (III) multiple-cell clones spanning the xylem, the cambium and the phloem; (IV) multiple-cell clones in the phloem; (V) single-cell clones in the phloem (Fig. 4A,B, Figs S5-S8).

The majority of the clones that originated from *PXY_pro_*- and *WOX4_pro_*-positive cells fell into category I, II or III (74/82, 90%). In contrast, all clones that originated from *SMXL5_pro_*-positive cells were in category III, IV or V (17/17, 100%) (Fig. 4B). This finding demonstrates that cells in the proximal cambium are determined for xylem development and cells in the distal cambium are determined exclusively for phloem development. Importantly, a large number of type-III clones were identified regardless of the site of initial induction (35/99, 35%) whereas only two clones (2/99, 2%) were found ranging from differentiated xylem or phloem cells to undifferentiated cambium cells. Assuming that single cells were the origin of most of the type-III clones, this strongly suggests the existence of one bifacial stem cell within each individual cell file that produces both xylem and phloem cells. This cell should be characterized by *PXY*, *WOX4* and *SMXL5* promoter activity and reside in the central cambium. In contrast, xylem or phloem mother cells contribute only for a short period to the respective tissue productions.

Collectively, we established a cambium-centered fate map during radial growth of the *Arabidopsis* hypocotyl (Fig. 4C). We determined that cells that originate from *PXY_pro_*- and *WOX4_pro_*-positive cells contribute to the region from X6 to P5 (Fig. 4B) and cells that originate from *SMXL5_pro_*-positive cells contribute to the region from X4 to P6, which suggests that tissues with a radial dimension of ∼500 µm originated from one cambium region over a period of 10 days. As the collection of identified cell clones formed a continuum from differentiated xylem to differentiated phloem with a high coverage rate, we were indeed able to target all stages of cells during their transition from cambium stem cells to differentiated vasculature.

Because type-III clones were clearly overrepresented among all clones identified, one *PXY*, *WOX4* and *SMXL5*-positive cambial stem cell (marked as triangle in Fig. 4C) appears to continuously proliferate and produce cells for both xylem and phloem production. Considering cases of a rather flexible boundary formation between two cellular domains (Caggiano et al., 2017) and a possible temporal fluctuation of cellular functions within the cambium (Johnsson and Fischer, 2016), it is also possible that this cell activates the respective promoters in a sequential order.

In both scenarios this bifacial stem cell continuously undergoes asymmetrical cell divisions in each radial cell file (Kajala et al., 2014) alternating between the production of xylem and phloem progenitors, as indicated recently by a wound-induced sector analysis in poplar (Bossinger and Spokevicius, 2018). Whether the two sister cells are equal immediately after division, and only one of both cells acquires xylem or phloem fate afterwards or whether bifacial stem cells harbor an intrinsic asymmetry transferred to daughter cells remains to be determined. In addition to asymmetrical cell divisions, our clonal analysis also suggests the occurrence of anticlinal divisions of stem cells, which are considered as symmetrical divisions that generate two cambium stem cells.

Interestingly, when determining the xylem/phloem proportion in each type-III clone, we observed a wide distribution (Fig. S9). Currently, it is unclear whether this variation represents a heterogeneity in stem cell function or reflects a rather stochastic dynamic of tissue production within the cambium.

Importantly, we could not detect EdU-positive nuclei remaining in the central cambium in our EdU incorporation experiment (Fig. 1D) and our clonal analysis also argues against the common existence of slowly dividing cells in that region. In animals, adult stem cells usually divide relatively slowly (Alberts et al., 2015; Fuchs, 2009) and in plants quiescence of central parts of stem cell regions is a fundamental property (Greb and Lohmann, 2016). Our findings imply that stem cell behavior during radial growth differs from those systems in this regard. A low division rate of stem cells compared with immediate descendants is considered to be important for maintaining the integrity of genetic information. In comparison with apical meristems, during radial growth a larger pool of stem cells produces a lower number of descendants. Because the effect of disturbed genetic integrity in individual stem cells is therefore also lower, this difference might explain why quiescence of cambial stem cells is not very pronounced.

## MATERIALS AND METHODS

### Plant materials

*Arabidopsis thaliana* (L.) Heynh. *Col-0* plants were used as a genetic background. Seeds were sterilized by brief washes with 70% ethanol supplemented with 0.1% Tween-20 (Carl Roth, 9127), sown on 0.8% agar in 1/2 Murashige and Skoog (MS) medium plates supplemented with 1% sucrose and then stratified in darkness at 4°C for 3 days. Seedlings were grown in long-day conditions (16 h light and 8 h darkness) at 21°C. The *WOX4_pro_:ER-YFP* line (*pPS11*) has been described previously (Suer et al., 2011). Other transgenic lines were generated through the floral dipping method using *Agrobacterium tumefaciens* (Clough and Bent, 1998).

### Vector construction

*pML988* was a gift from Michael Lenhard (Anastasiou et al., 2007). An H4-GFP-containing construct was a gift from Daniel Schubert (Freie Universität Berlin, Germany). A histone 2B-RFP-containing construct was a gift from Alexis Maizel [The Centre for Organismal Studies (COS) Heidelberg, Germany]. *PXY_pro_:H4-GFP* (*pPS24*), *SMXL5_pro_:H4-GFP* (*pIL53*), *WOX4_pro_:H4-GFP* (*pPS04*) was cloned using the *pGreen0229* vector (Hellens et al., 2000) as a backbone. Cloning of *WOX4* and *PXY* promoter and terminator regions was described previously (Agusti et al., 2011a; Suer et al., 2011). The *SMXL5* promoter region was cloned using *CEB1for11* and *CEB1rev2* primers, and the *SMXL5* terminator region was cloned using *CEB1for3* and *CEB1rev3* primers. The *H4-GFP* fragment was cloned into a vector carrying the *PXY* promoter and terminator regions using *H4GFP-PXYfor* and *H4GFP-APLrev* primers, into a vector carrying the *SMXL5* promoter and terminator regions using *H4GFP_for1* and *H4GFP_rev1* primers, and into a vector carrying the *WOX4* promoter and terminator regions using *H4GFP-APLfor* and *H4GFPWOX4rev* primers. Other plasmids were generated using the GreenGate cloning system (Lampropoulos et al., 2013; Schurholz et al., 2018). See Table S2 for each module used during the cloning process. The *Cre* and *H2B-RFP* fragment was cloned into an entry vector (pGGI000) using *Cre-pGGI000-5-TG495* and *NewCre-pGGI000-3* primers (*pDS03*), and *H2BRFPinpGGI-Fwd* and *H2BRFPinpGGI-Rev* primers (pDS77), respectively. Primer sequences are indicated in Table S3.

### EdU incorporation and detection

Plants were kept on agar until the day mentioned. Plants were cropped together with some agar, and transferred into liquid 10 µM EdU 1/2 MS medium. For pulse-chase experiments (Fig. 1B), plants were kept in the EdU medium for 2 days and then transferred to soil. For proliferation assays (Figs 1C,D, 2C-H), plants were incubated in the EdU medium for 6 h, washed with 1/2 MS, and incubated for 90 h in liquid 1/2 MS medium and then transferred to soil. Under this regime, we assume that EdU is incorporated for 4 days in total as the agar retains EdU even after one medium change, and this protocol was intended to ensure thorough labeling of proliferative cells. Indeed, the EdU signal was detected in all plants that were analyzed, whereas the pulse-chase regime used for experiments described in Fig. 1 resulted in EdU incorporation only in a fraction of the plants, as previously reported (Watson et al., 2016). Plants were collected at the time described and hypocotyls were dissected and fixed using 4% paraformaldehyde (PFA) in Ca^2+^, Mg^2+^-free PBS at 4°C overnight. After washing with Ca^2+^, Mg^2+^-free PBS, samples were embedded in warm 7% low melting temperature agarose (Sigma-Aldrich, A4018). After solidification, 200 µm sections were made using a vibratome (Leica, VT1000 S), and the sections embedded in agarose were stained according to the manufacturer's protocol (Thermo Fisher Scientific, Click-iT EdU Imaging Kits Alexa Fluor 594, 10339). Nuclei were stained using 4′,6-diamidino-2-phenylindole (DAPI). Lignified cell walls in the differentiated xylem are visualized by auto-fluorescence using the 405 nm laser.

### Dex induction

*PXY_pro_:LhGR-N;Op4_pro_:Cre*, *SMXL5_pro_:LhGR-N;Op4_pro_:Cre* and *WOX4_pro_:LhGR-N;Op4_pro_:Cre* T2 plants were crossed with a homozygous *pML988* plant line. F1 seeds were obtained and used for Dex induction experiments. For *NST3_pro_:LhGR-N;Op4_pro_:Cre;;pML988* and *APL_pro_:LhGR-N;Op4_pro_:Cre;;pML988* plants, *NST3_pro_:LhGR-N;Op4_pro_:Cre* or *APL_pro_:LhGR-N;Op4_pro_:Cre* transgenes were transformed to a homozygous *pML988* plant line. T2 seeds were obtained and used for Dex induction experiments.

Plants were selected based on Hygromycin B resistance on vertical 1% agar 1/2 MS plates (Harrison et al., 2006) and then transferred to soil. At 22 dag, a tissue (Kimtech Science) was tied around the hypocotyl. Dex (BioChemica, A2153) was dissolved in DMSO, and diluted in tap water with 0.02% Silwet L-77. Then 500 µl of the diluted solution of Dex was applied to the paper knot. This paper knot was removed from the plants one day after the induction for the initial analyses (Fig. 3, Fig. S4) and 5 min after the induction for the individual clonal analysis (Fig. 4). The final Dex concentration was 500 nM (*PXY_pro_*) and 2.5 μM (*SMXL5_pro_*, *WOX4_pro_*, *NST3_pro_*, *APL_pro_*) for experiments described in Fig. 3 and Fig. S3, and 100 nM (*PXY_pro_*) and 500 nM (*SMXL5_pro_*, *WOX4_pro_*) for experiments described in Fig. 4. Hand sections of the hypocotyl were analyzed as described in the microscopy section. Samples with no fluorescence detected, probably because of the incomplete Dex penetration, were discarded.

### Microscopy

Free-hand sections of hypocotyls were made by razor blades (Wilkinson Sword) and put into a glass-bottom dish (ibidi, µ-Dish 35 mm, high, 81151). Images were captured using a Nikon A1 confocal microscope with a ×25 water immersion objective lens (Nikon, Apo 25xW MP, 77220) and gallium arsenide phosphide (GaAsP) detectors or normal photomultiplier detectors. Then 405 nm, 457 nm, 514 nm and 561 nm lasers were used to excite DAPI/Xylem (auto-fluorescence), CFP, YFP, Direct Red/Alexa Fluor 594, respectively. In the multichannel images of YFP and Direct Red 23, YFP was excited by a 488 nm laser and a 540/30 filter was used to collect emission fluorescence. For Direct Red 23 staining (Anderson et al., 2010; Ursache et al., 2018; Wallace and Anderson, 2012), free-hand sections of hypocotyls were put directly into 0.1% solution of Direct Red 23 (30% content powder, Sigma-Aldrich, 212490) in Ca^2+^, Mg^2+^-free PBS for more than 20 min. After brief washing with tap water, images were captured. For images in Fig. 2B, hypocotyls were fixed with 4% PFA and sections were made using a vibratome as described in the EdU incorporation and detection section. The images shown in Fig. S2 were captured by Leica SP5 confocal microscope with a ×20 water immersion objective lens (Leica, HCX PL APO lambda blue 20.0×0.70 IMM UV). Also, 514 nm and 561 nm lasers were used to excite YFP and Direct Red, respectively, and emission was collected in the 520-555 nm and 570-620 nm ranges, respectively. The images shown in Fig. 2A were captured using a Leica SP8 confocal microscope with a glycerol immersion objective lens (Leica, HC PL APO 63×/1.30 Glyc CORR CS2) after embedding into an agarose block and sectioning using a vibratome as described in the EdU incorporation and detection section. Then 458 nm, 514 nm and 561 nm lasers were used to excite CFP, YFP and Direct Red, respectively, and emission was collected in the 465-509 nm, 519-584 nm and 599-692 nm ranges, respectively. The region of differentiated xylem is defined by the enlarged cell size, which is visible under Direct Red 23 staining or transmitted images.

### Intensity profile analysis

We cropped and used maximum intensity projection on 150×450 pixel (Fig. 1B) or 150×400 pixel (Fig. 2C-H) regions of images around the cambium. Contrast and brightness adjustment was applied to the whole area of the image to eliminate the week auto-fluorescence of xylem cell walls. The same adjustment was applied for all images of each analysis. Intensities of each channel was projected to the *y*-axis, and then averaged using multiple images (see figure legends). The maximum intensity of each channel across the *y*-axis was normalized to 1. This analysis was carried out using Fiji (Schindelin et al., 2012) and Excel (Microsoft). Cross-correlation analysis was carried out in R using the ‘ccf’ function.

## Supplementary Material

Supplementary information
