# Supplementary material for: Bifacial cambium stem cells generate xylem and phloem during radial plant growth
Source: Development. 2019 Jan 9;146(1):dev171355. doi: 10.1242/dev.171355 (PMC6340147; doi:10.1242/dev.171355)
Supplement: Supplementary information [file develop-146-171355-s1.pdf]

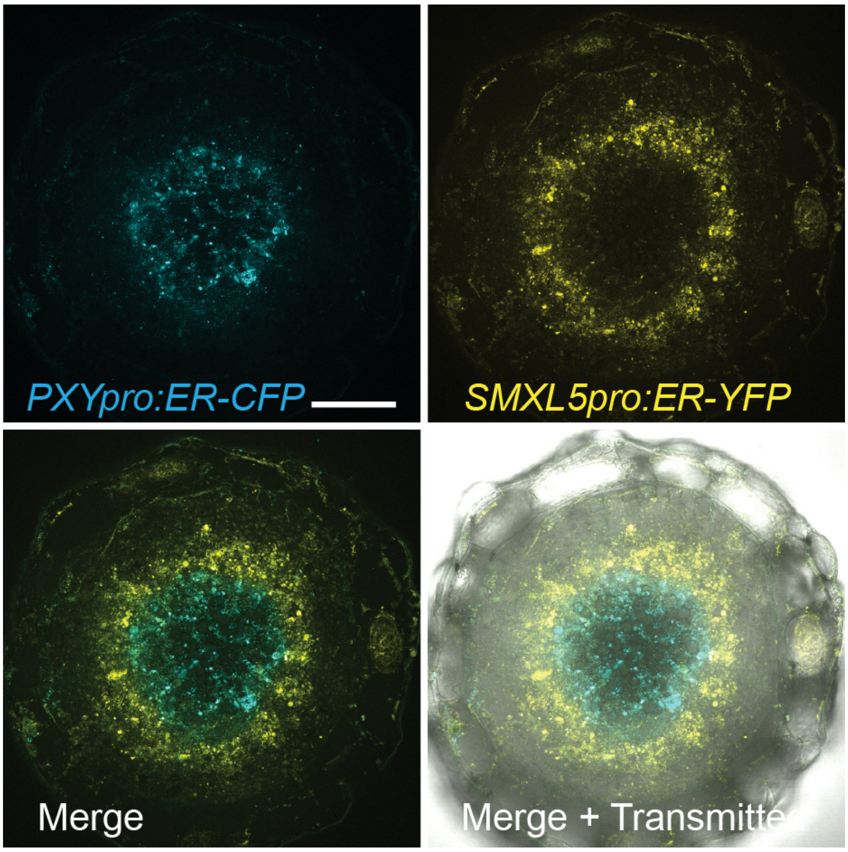

Fig. S1. Characterization of *PXY/TDR* and *SMXL5* promoter activities.
Maximum intensity projection of confocal images of *PXYpro:CFP;SMXL5pro:YFP* hypocotyl cross sections at 21 dag. Size = 50 µm. n = 2.

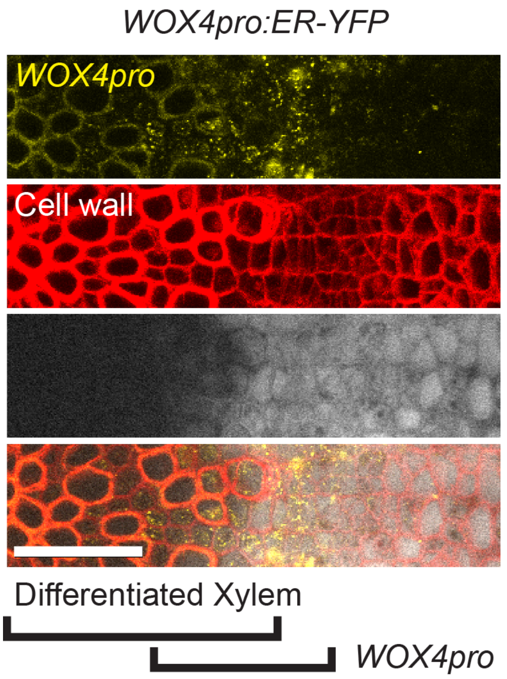

Fig. S2. Characterization of *WOX4* promoter activities.

Maximum intensity projection of confocal images of a hypocotyl cross section from a *WOX4$_{pro}$:ER-YFP* marker line at 21 dag. Cell walls are visualized by Direct Red 23 (in red). Size = 50 µm. The hypocotyl center is located left.

Development • Supplementary information

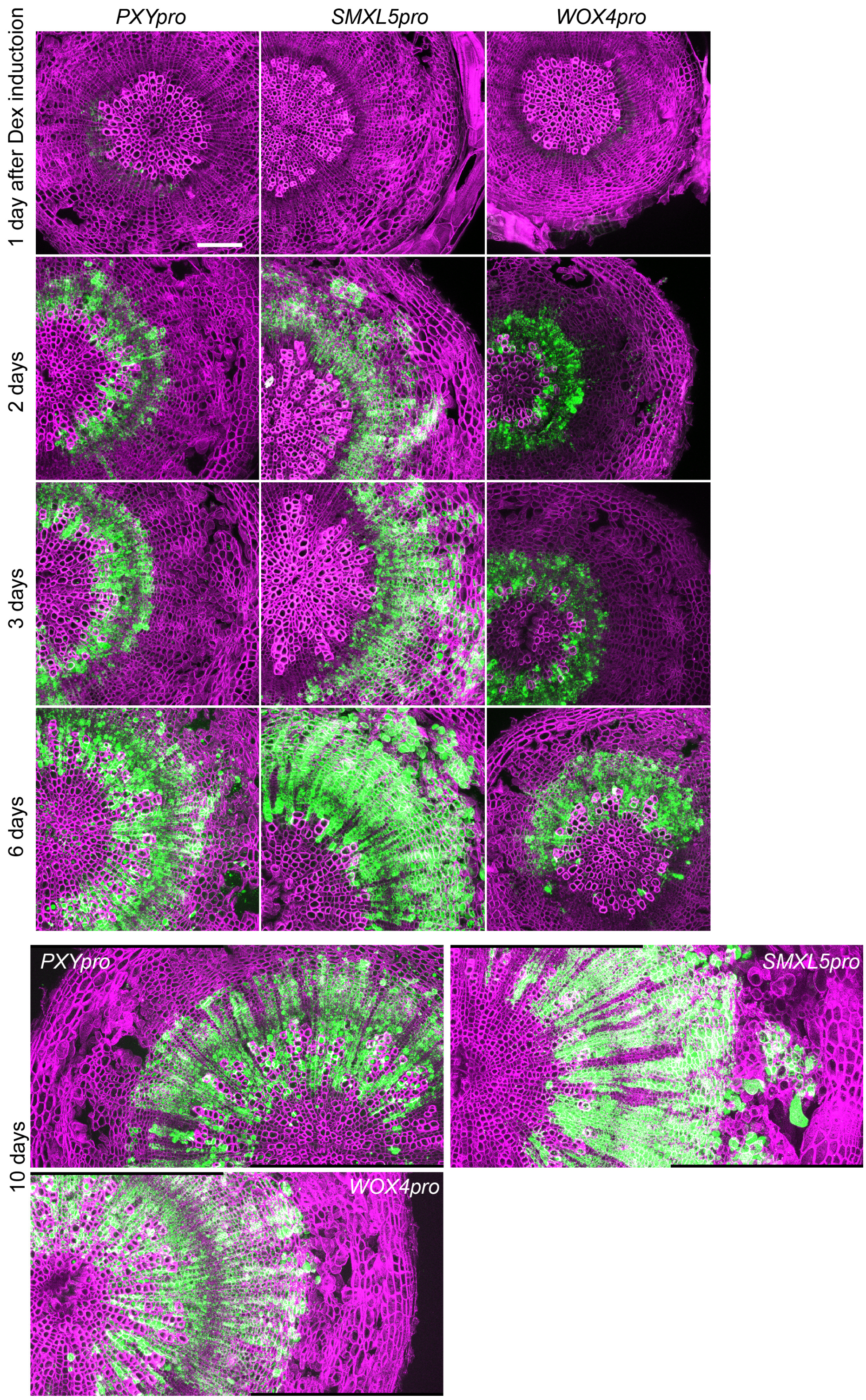

Fig. S3. Full images of cell lineage analyses shown in Fig. 3.

Maximum intensity projections of confocal images of cross sections of Dex-induced hypocotyls. *PXY_{pro}:LhGR-N;$Op4_{pro}$:Cre;;pML988* (labeled as *PXY_{pro}*), *$SMXL5_{pro}$:LhGR-N;$Op4_{pro}$:Cre;;pML988* (*$SMXL5_{pro}$*) and *$WOX4_{pro}$:LhGR-N;$Op4_{pro}$:Cre;;pML988* (*$WOX4_{pro}$*) plant lines were treated with Dex at 22 dag by local application. YFP signals are shown in green and cell walls are visualized by Direct Red 23 (shown in magenta). Size bar = 100 µm. Same magnification in all images.

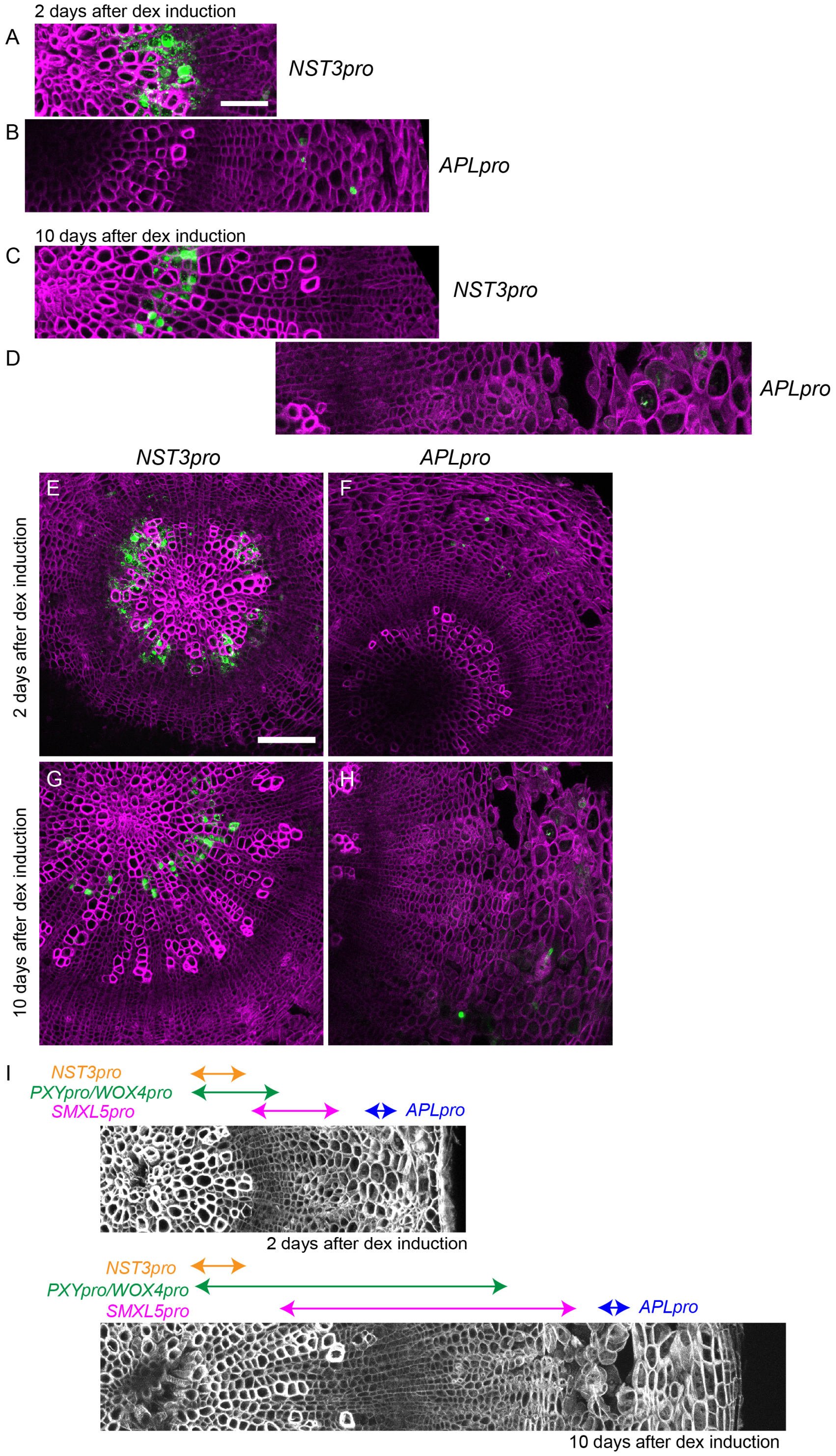

Fig. S4. Cell lineage tracing of *NST3, APL*-positive cells.

(A–H) Maximum intensity projection of confocal images of cross sections of Dex-induced hypocotyls. *NST3pro:LhGR-N;Op4pro:Cre;;pML988* (labeled as *NST3pro*), *APLpro:LhGR-N;Op4pro:Cre;;pML988* (labeled as *APLpro*). YFP activity is shown in green, and cell walls are visualized by Direct Red 23 staining (magenta). Images of 2 days (A, B) and 10 days (C, D) after Dex induction, respectively, are shown. Uncropped images of A–D are shown in E–H, respectively. Size bar = 50 μm (A–D), 100 μm (E–H). n = 3, 6, 4, 6 for A–D, respectively. (I) Schematic summary for the position of labeled cells in the lineage tracing analysis of each promoter. 2 days (top), or 10 days (bottom) after Dex induction.

Development • Supplementary information

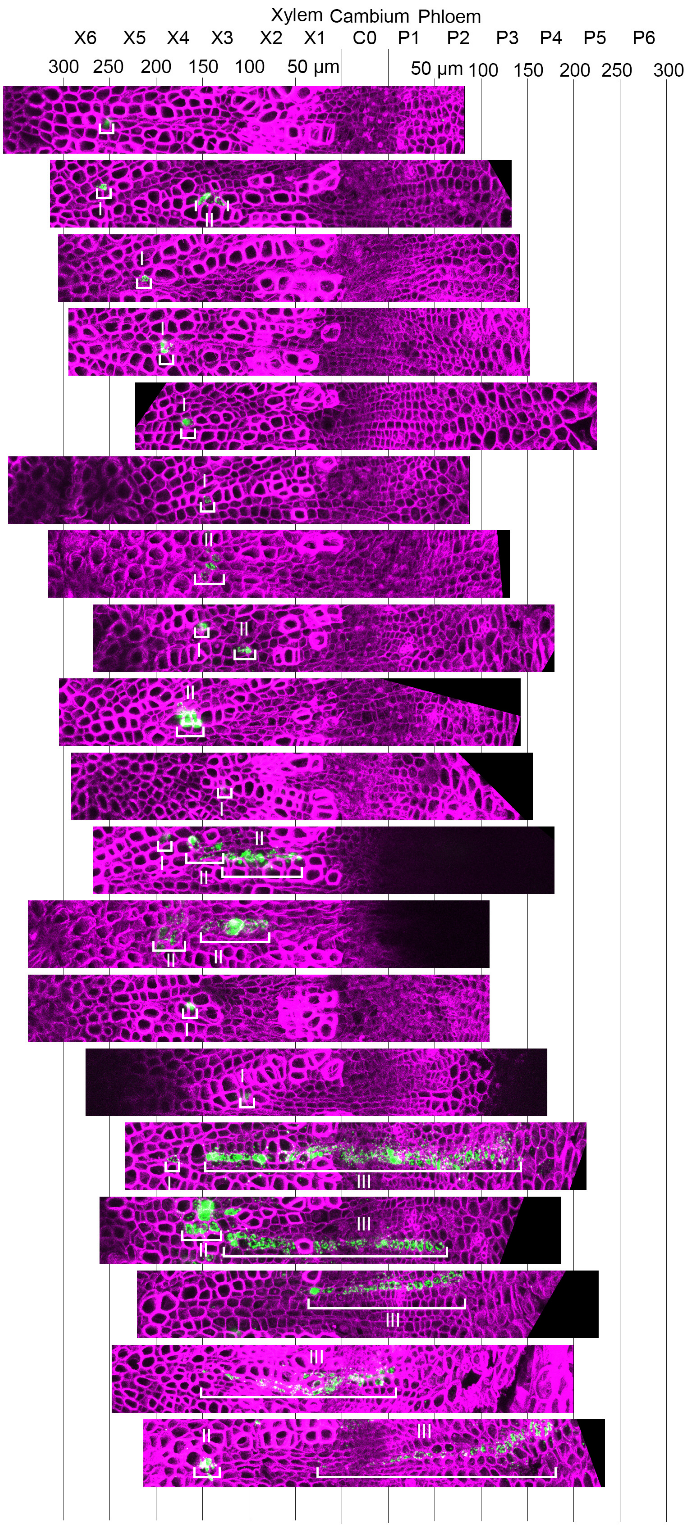

Fig. S5. Morphology of cell clones having originated from *PXY*$_{pro}$-positive cells. Maximum intensity projection of confocal images of hypocotyl cross sections. YFP signals are shown in green and cell walls are visualized by Direct Red 23 and shown in magenta. The hypocotyl center is located left. White brackets indicate the position of the YFP-positive clone. Clones were categorized into “I” to “V” according to their localization and morphology as shown in Fig. 4B.

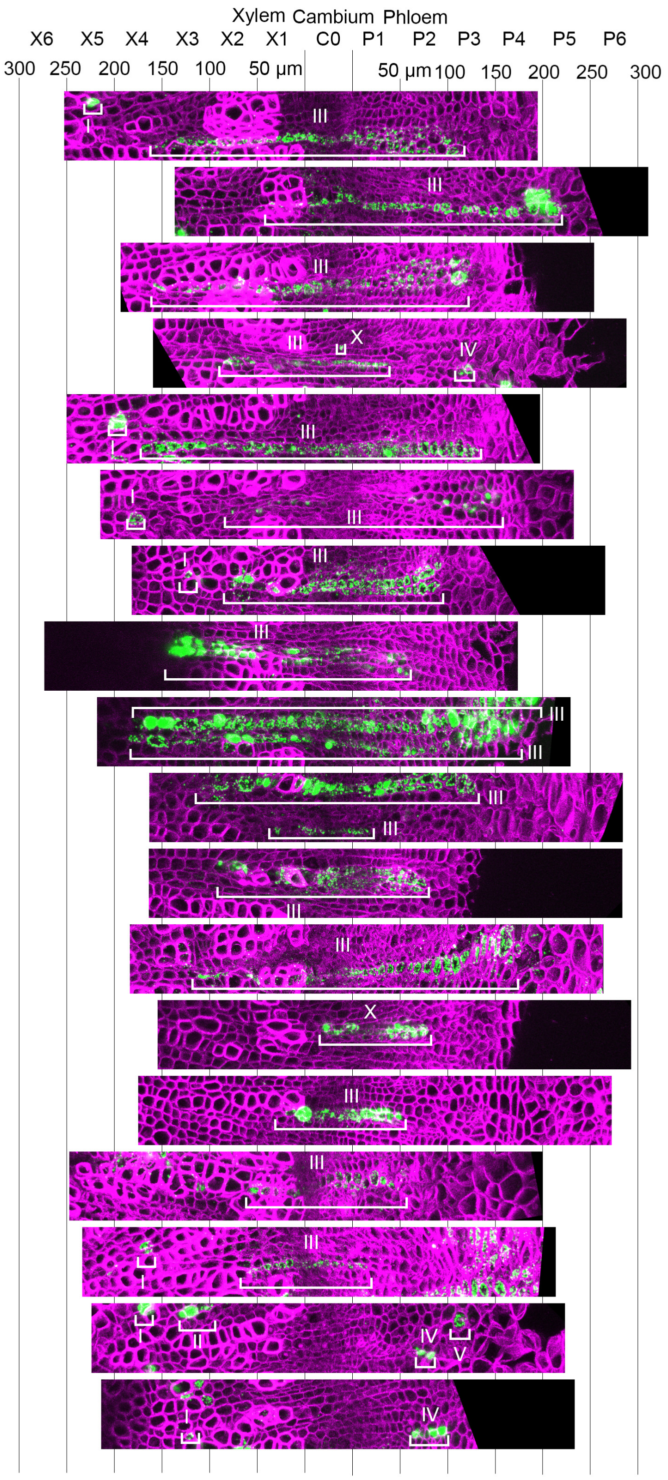

Fig. S6. Morphology of cell clones having originated from *PXY*$_{pro}$-positive cells. Maximum intensity projection of confocal images of hypocotyl cross sections. YFP signals are shown in green and cell walls are visualized by Direct Red 23 and shown in magenta. The hypocotyl center is located left. White brackets indicate the position of the YFP-positive clones. Clones were categorized into “I” to “V” according to their localization and morphology as in Fig. 4B. Clones labeled with “X” did not fit any category.

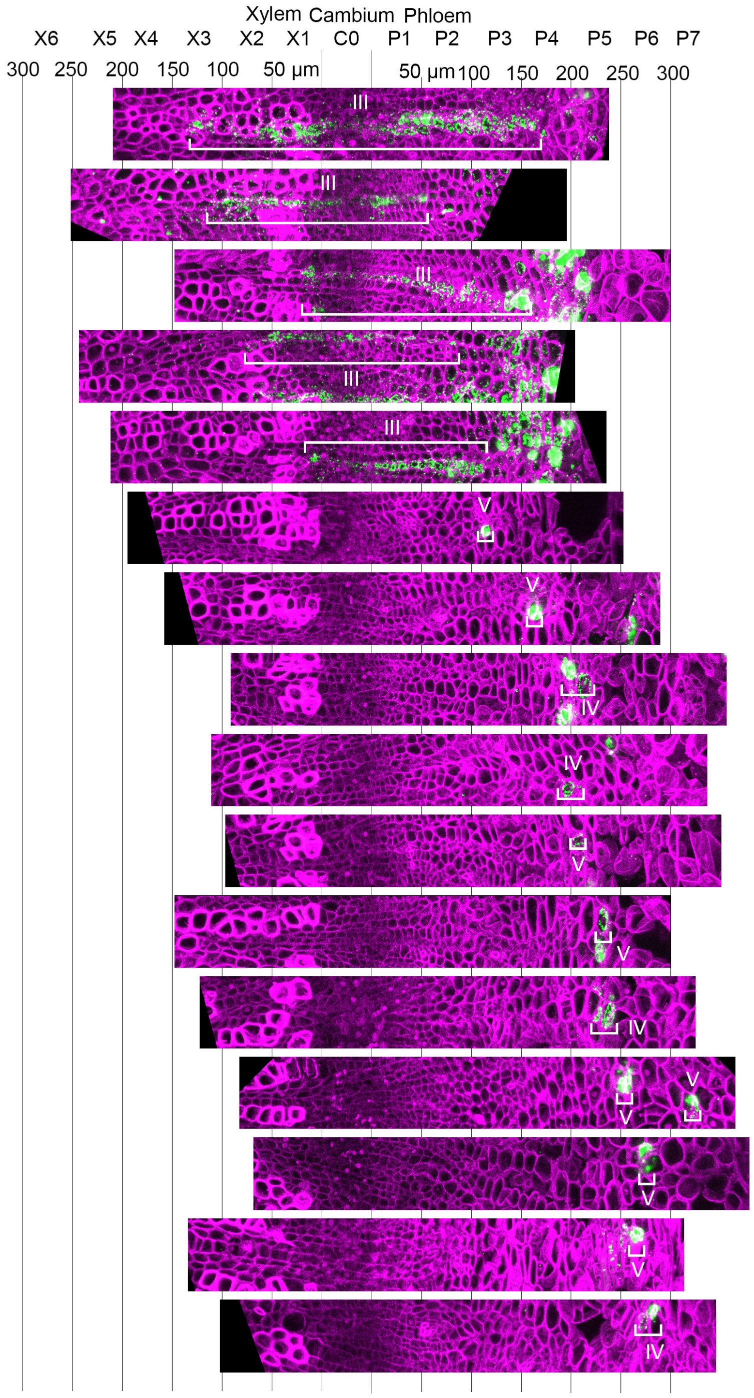

Fig. S7. Morphology of cell clones having originated from *SMXL5$_{pro}$*-positive cells. Maximum intensity projection of confocal images of hypocotyl cross sections. YFP signals are shown in green and cell walls are visualized by Direct Red 23 and shown in magenta. The hypocotyl center is located left. White brackets indicate the position of cell clones. Clones were categorized into “I” to “V” according to their localization and morphology as in Fig. 4B.

Development • Supplementary information

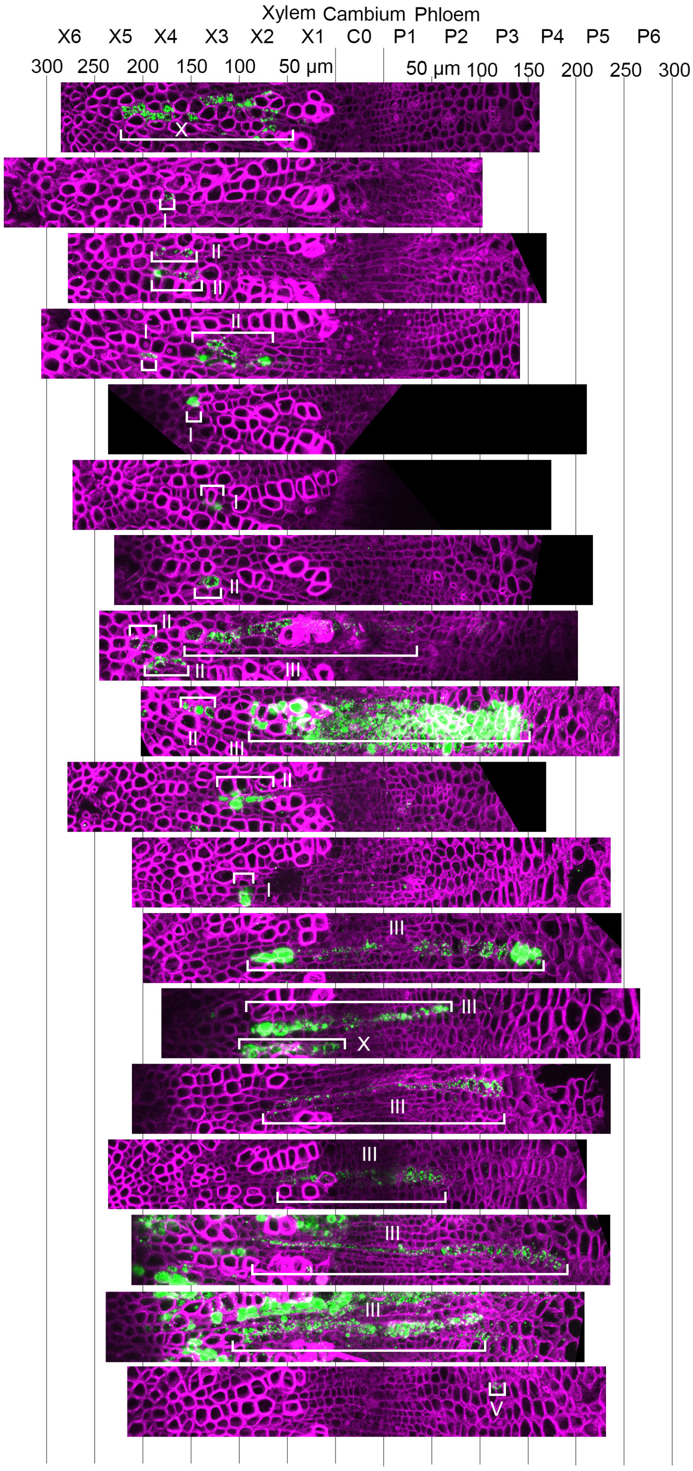

Fig. S8. Morphology of cell clones having originated from *WOX4$_{pro}$*-positive cells. Maximum intensity projection of confocal images of hypocotyl cross sections. YFP signals are shown in green and cell walls are visualized by Direct Red 23 and shown in magenta. The hypocotyl center is located left. White brackets indicate the position of the cell clones. Clones were categorized into “I” to “V” according to their localization and morphology as in Fig. 4B. Clones labeled with “X” did not fit any category.

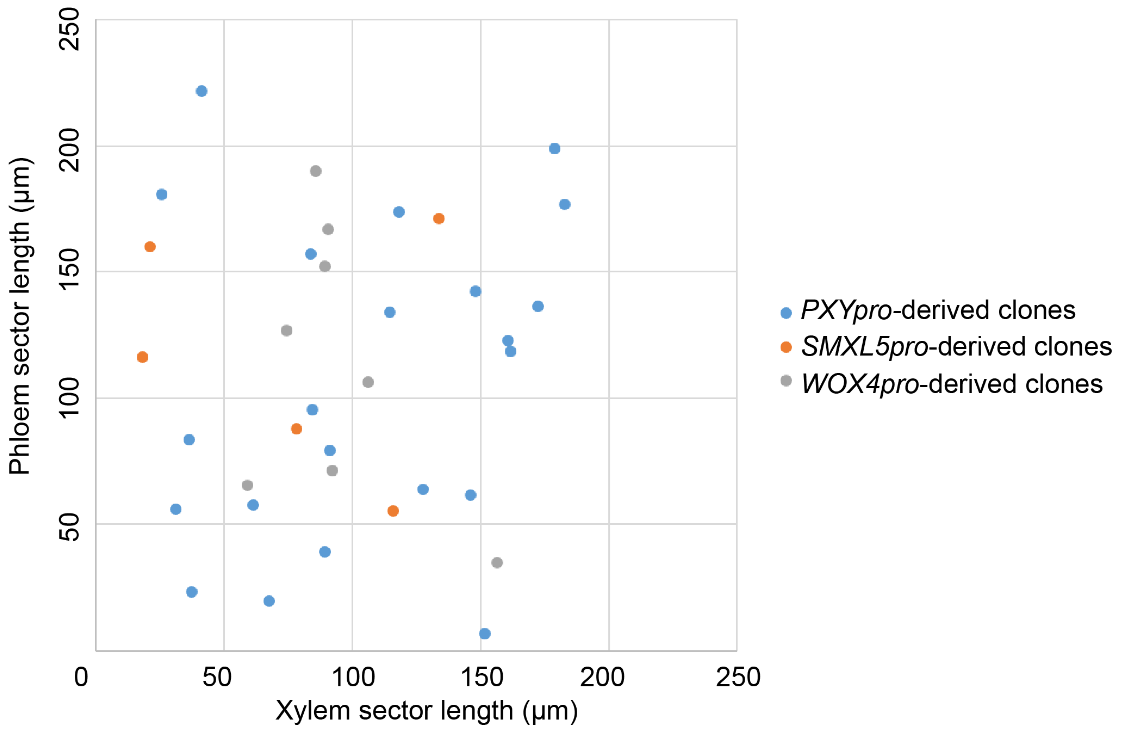

Fig. S9. Xylem / phloem sector length measurement of type III individual clones originated from *PXYpro*, *SMXL5pro*, *WOX4pro*-positive cells.

The xylem or phloem sector length was measured for each type III individual clones shown in Fig. 4B (also in Fig. S5, S6, S7, S8). Xylem or phloem were identified using the grid shown in 4B where the boundary between xylem and cambium is set by the auto-florescence of lignified cell wall, and cambium region is set to have 50 µm width in the section image. n = 22 (*PXYpro*), 5 (*SMXL5pro*), 8 (*WOX4pro*) clones.

Development • Supplementary information

Table S1. Cross correlation analysis between EdU signal profiles from different time points.

	Day 0	Day 2	Day 4	Day 6	Day 8	Day 10	Day 12
Day 0	1.000						
Day 2	0.496	1.000					
Day 4	0.361	0.115	1.000				
Day 6	-0.202	0.055	0.018	1.000			
Day 8	-0.328	-0.283	-0.013	0.309	1.000		
Day 10	-0.181	-0.045	-0.065	0.558	0.350	1.000	
Day 12	-0.273	-0.210	-0.197	0.151	0.066	0.148	1.000

The EdU profiles from different time points after EdU incorporation shown in Fig. 1B are used for cross correlation analysis. Correlation values at lag = 0 pixel, obtained from pair-wise comparison, are shown in the table. Value lower than 0.3 are highlighted in yellow, as it suggests that the compared two data set have little correlation.

Table S2. GreenGate Vectors used in this study.

Vector name	Vector ID	Modules
PXY_{pro}:ER-CFP; $SMXL5_{pro}$:ER-YFP	pVL78	PXY_{pro} (pVL27*), ER Signal Peptide (pGGB006†), mTurquoise2 (pSW596*), HDEL (pGGD008†), tPXY (pVL29*), F-H adapter (pGGG001†), H-A adapter (pGGG002†), $SMXL5_{pro}$(pVL28*), ER Signal Peptide (pGGB006†), VENUS (pSW549*), HDEL (pGGD008†), tSMXL5 (pVL20*), BastaR (pGGF001†), Destination vector (pGGZ003†)
$SMXL5_{pro}$:H2B-RFP	pDS77	$SMXL5_{pro}$ (pVL28*), H2B-RFP (pDS76), tSMXL5 (pVL20*), HygroR (pGGF012†), Destination vector (pGGZ003†)
PXY_{pro}:LhGR-N; $Op4_{pro}$:Cre	pDS26	PXY_{pro} (pVL27*), GR-LHG4_BD (pSW610*), tPXY (pVL29*), F-H adapter (pGGG001†), H-A adapter (pGGG002†), $Op4_{pro}$ (pSW180*), Cre (pDS03), tUBQ10 (pGGE009†), HygroR (pGGF005†), Destination vector (pGGZ003†)
$SMXL5_{pro}$:LhGR-N; $Op4_{pro}$:Cre	pDS27	$SMXL5_{pro}$ (pVL28*), GR-LHG4_BD (pSW610*), tSMXL5 (pVL20*), F-H adapter (pGGG001†), H-A adapter (pGGG002†), $Op4_{pro}$ (pSW180*), Cre (pDS03), tUBQ10 (pGGE009†), HygroR (pGGF005†), Destination vector (pGGZ003†)
$WOX4_{pro}$:LhGR-N; $Op4_{pro}$:Cre	pDS28	$WOX4_{pro}$ (pVL37*), GR-LHG4_BD (pSW610*), tWOX4 (pVL22*), F-H adapter (pGGG001†), H-A adapter (pGGG002†), $Op4_{pro}$ (pSW180*), Cre (pDS03), tUBQ10 (pGGE009†), HygroR (pGGF005†), Destination vector (pGGZ003†)
$NST3_{pro}$:LhGR-N; $Op4_{pro}$:Cre	pDS70	$NST3_{pro}$ (pVL18*), GR-LHG4_BD (pSW610*), tNST3 (pVL13*), F-H adapter (pGGG001†), H-A adapter (pGGG002†), $Op4_{pro}$ (pSW180*), Cre (pDS03), tUBQ10 (pGGE009†), HygroR (pGGF005†), Destination vector (pGGZ003†)
APL_{pro}:LhGR-N; $Op4_{pro}$:Cre	pDS71	APL_{pro} (pVL35*), GR-LHG4_BD (pSW610*), tAPL (pVL25*), F-H adapter (pGGG001†), H-A adapter (pGGG002†), $Op4_{pro}$ (pSW180*), Cre (pDS03), tUBQ10 (pGGE009†), HygroR (pGGF005†), Destination vector (pGGZ003†)

* reference: Schuerholz et al., 2018
† reference: Lampropoulos et al., 2013

Development • Supplementary information

Table S3. Primers used in this study.

Primer name	Sequence (5‘-3‘)	Usage
CEB1for11	ACTAGGTACCCTTAGATTCGCACTCGCTCAACAACCGTGAGCTCTGA	SMXL5 promoter
CEB1rev2	ACTAGGATCCATCATATGAACTTGTAAACCCTAACCCTAAC	SMXL5 promoter
CEB1for3	ACTAGGATCCATCTGCAGTAGAATCTTTCGGTTAAATTTC	SMXL5 terminator
CEB1rev3	ACTAGCGGCCGCTCTTGTTAACTTTCCAAAATTTG	SMXL5 terminator
H4GFP-PXYfor	ACTAGCGGCCGCATGTCGGGTCGTGGAAAGGGA	PXY_{pro}:H4-GFP
H4GFP-APLrev	ACTACTGCAGTTATTTGTATAGTTCATCCATGC	PXY_{pro}:H4-GFP
H4GFP-APLfor	ACTAACATGTCGGGTCGTGGAAAGGGA	$WOX4_{pro}$: H4-GFP
H4GFPWOX4rev	ACTAGGATCCTTATTTGTATAGTTCATCCATGC	$WOX4_{pro}$: H4-GFP
H4GFP_for1	TATGTCGGGTCGTGGAAAGGGA	$SMXL5_{pro}$: H4-GFP
H4GFP_rev1	TGCATTATTTGTATAGTTCATCCATGC	$SMXL5_{pro}$: H4-GFP
Cre-pGGI000-5-TG495	AACAGGTCTCAAACAATGCATCTGGATCAATCCAATTTAC	Cloning of Cre
NewCre-pGGI000-3	AACAGGTCTCAGCAGCTAAATCGCCATCTTCCAGCAGG	Cloning of Cre
H2BRFPinpGGI-Fwd	AACAGGTCTCAAACAATGGCGAAGGCAGATAAGAAAC	Cloning of H2B-RFP
H2BRFPinpGGI-Rev	AACAGGTCTCAGCAGTTATAATTAGGCGCCGGTGG	Cloning of H2B-RFP
